# Development and Optimization of 7,8-Dihydroxyflavone-Loaded Polylysine/Lecithin Nanoparticles for Potential Intranasal Delivery

**DOI:** 10.3390/pharmaceutics18070766

**Published:** 2026-06-23

**Authors:** Sonya Salamone, Rosalia Pellitteri, Ilaria Ottonelli, Elide Zingale, Cinzia Cimino, Barbara Ruozi, Teresa Musumeci, Rosario Pignatello

**Affiliations:** 1 PhD Program in Neuroscience, Department of Biomedical and Biotechnological Sciences, School of Medicine, University of Catania, 95125 Catania, Italy; sonya.salamone@phd.unict.it; 2 Department of Drug and Health Sciences, University of Catania, 95125 Catania, Italy; elide.zingale@unict.it (E.Z.); cinzia.cimino@unict.it (C.C.); rosario.pignatello@unict.it (R.P.); 3 NANOMED—Research Centre on Nanomedicine and Pharmaceutical Nanotechnology, University of Catania, 95123 Catania, Italy; 4 Institute for Biomedical Research and Innovation, National Research Council, 95126 Catania, Italy; rosaliamariacristina.pellitteri@cnr.it; 5 NanotechLab, Traditional and Innovative Pharmaceutical Technologies Center, (Te.Far.T.I.), Department Life Sciences, University of Modena and Reggio Emilia, Via Campi 103, 41125 Modena, Italy; ilaria.ottonelli@unimore.it (I.O.); barbara.ruozi@unimore.it (B.R.)

**Keywords:** flavonoids, DoE, fish oil, mucin, DPPH, TEM

## Abstract

**Background**: Effective strategies for delivering neuroprotective agents to the brain remain a major challenge due to the poor solubility, rapid metabolism, and low bioavailability of promising molecules, such as 7,8-dihydroxyflavone (7,8-DHF). This small-molecule TrkB receptor agonist exhibits significant antioxidant, neuroprotective properties, and additional effects on metabolic regulation, but its therapeutic potential is limited by unfavorable pharmacokinetic characteristics. Nanotechnology-based delivery systems are increasingly explored to improve drug stability, enhance bioavailability, and facilitate direct nose-to-brain transport following intranasal administration. In this study, lipid nanoparticles encapsulating 7,8-DHF were developed using a fish-oil-based lipid core enriched with ω-3 polyunsaturated fatty acids (DHA and EPA) and naturally derived excipients, including soybean lecithin and ε-polylysine. **Methods**: The formulation was optimized using a Design of Experiments (DoE) approach based on a 2^3^ full factorial design, evaluating drug concentration, lecithin concentration, and surfactant type (Pluronic^®^ F127 or Tween^®^ 80). The main formulation responses considered were particle size, polydispersity index (PDI), zeta potential, and encapsulation efficiency. **Results**: The optimized nanoparticles exhibited nanometric dimensions (<250 nm); spherical morphology, confirmed by TEM; low polydispersity (PDI < 0.3); and adequate encapsulation efficiency. Stability studies in simulated biological fluids indicated good physicochemical stability for up to 48 h, while interaction studies with mucin suggested a good interaction within the mucus environment. ROS scavenging capacity was confirmed through the DPPH chemical assay, and in vitro experiments on olfactory ensheathing cells, selected as a biologically relevant model for their anatomical localization along the olfactory pathway, showed reduced cytotoxicity of the encapsulated drug compared with the free form. **Conclusions**: Collectively, these results support the potential application of the developed nanoformulation in the intranasal delivery of 7,8-DHF.

## 1. Introduction

Neurodegenerative and metabolic disorders represent a major global health burden, with rising incidence rates and an urgent need for innovative therapeutic strategies. Growing epidemiological and preclinical evidence suggests that these conditions may share common molecular underpinnings, including chronic inflammation, oxidative stress, and insulin resistance, raising interest in dual-action therapeutic approaches capable of simultaneously targeting peripheral metabolic dysfunction and central neuronal integrity [1,2,3,4]. Among the various molecular candidates under investigation, 7,8-dihydroxyflavone (7,8-DHF), a naturally occurring flavonoid, is attracting growing attention. Acting as a selective small-molecule agonist of the TrkB receptor, the principal receptor for brain-derived neurotrophic factor (BDNF), 7,8-DHF has demonstrated promising neuroprotective, cognitive-enhancing, and anorexigenic effects across a range of preclinical models [5,6,7].

Flavonoids are a large class of plant-derived polyphenolic compounds widely distributed in fruits, vegetables, seeds, leaves, and agro-industrial by-products. Structurally, they share a common C6–C3–C6 backbone comprising two aromatic rings linked by a heterocyclic pyran ring, which allows extensive substitution patterns and contributes to their diverse biological activities. Owing to their antioxidant, anti-inflammatory, anticancer, antiviral, and vasoprotective properties, flavonoids have attracted considerable attention for nutraceutical and pharmaceutical applications [8,9].

Although 7,8-DHF is already marketed in oral supplement form, its classification as a nutraceutical rather than a drug reflects the lack of clinical data supporting efficacy and pharmacokinetic reliability. The poor aqueous solubility, rapid metabolism, and low oral bioavailability (≈4–5%) of 7,8-DHF result in suboptimal systemic exposure and minimal brain concentrations after conventional administration [10,11]. These limitations remain major barriers to its translation into clinical use despite its favorable safety profile suggested by preclinical data and continued commercial availability [12]. These drawbacks highlight the need for innovative strategies to improve the pharmacological profile of 7,8-DHF and enhance its therapeutic applicability. A promising approach involves the application of nanomedicine, which can protect 7,8-DHF from light degradation, enhance its solubility, and thus ameliorate its pharmacokinetic profile [13,14,15]. When combined with intranasal administration, nanoparticle-based systems offer the additional advantage of bypassing both the blood–brain barrier and hepatic first-pass metabolism. By exploiting olfactory and trigeminal pathways, nose-to-brain delivery enables direct drug transport to the central nervous system, circumventing the pharmacokinetic bottlenecks that limit conventional systemic routes [16,17].

In this study, a natural nanoparticle formulation was developed with the aim of enhancing both delivery efficiency and biological relevance. The formulation employs naturally derived excipients that, to the best of our knowledge, have not previously been combined for 7,8-DHF encapsulation, resulting in a distinct composition compared with existing systems.

A key feature of this approach is the use of a fish-oil-based lipid core in place of typical neutral oils. Rich in ω-3 polyunsaturated fatty acids, such as EPA and DHA, fish oil provides intrinsic antioxidant, anti-inflammatory, and neuroprotective properties, potentially acting synergistically with 7,8-DHF [18,19]. In this context, the oily phase serves not only as a vehicle but as a bioactive component, contributing functionally to the overall neuroprotective effect.

Such a system may be particularly advantageous for intranasal administration, where safety and mucosal tolerability are critical. The present work therefore describes the formulation, physicochemical characterization, and preliminary in vitro assessment of these 7,8-DHF-loaded nanoparticles as a potential intranasal delivery system.

A rational formulation strategy was employed, guided by a Design of Experiments (DoE) approach, to systematically investigate the effects of formulation variables on nanoparticle performance. The selected critical quality attributes (CQAs) included mean particle size, polydispersity index (PDI), zeta potential, and encapsulation efficiency (%EE). This approach aligns with the principles of Formulation by Design (FbD), which complements Quality by Design (QbD), by enabling the rational development of advanced delivery systems through structured experimental design, risk assessment, and multivariate statistical analysis [20]. Like previously reported strategies in nanoparticle formulation, DoE and FbD frameworks allow precise control over formulation parameters and facilitate optimization of key quality attributes while minimizing experimental effort.

Given the intended intranasal administration route, we investigated the stability of the nanocarriers under physiological conditions. The optimized formulation was deeply characterized by its physicochemical properties. Furthermore, we demonstrate the potential for in vivo delivery of 7,8-DHF without organic solvents and show that encapsulation significantly reduces in vitro cytotoxicity.

## 2. Materials and Methods

### 2.1. Materials

7,8-Dihydroxyflavone (7,8-DHF); soybean lecithin; poloxamer 407; Tween 80^®^; 2,2-Diphenyl-1-picrylhydrazyl (DPPH); mucin (mucin from porcine stomach type II); NaCl; CaCl_2_·2H_2_O; KCl; NaH_2_PO_4_ · 7H_2_O; MgCl_2_ · 6H_2_O; CaCl_2_ · 2H_2_O; Na_2_HPO_4_; ethanol; and acetone were purchased from Merck (Milan, Italy). Epsiliseen^®^-H was a gift from Avg Srl (Bollate, Milan, Italy), a locally exclusive distributor of Epsiliseen^®-^H manufacturer Siveele B.V., (Breda, the Netherlands). Purified fish oil was generously gifted by Lipoid AG (Steinhausen, Switzerland) and distributed by Avg Srl (Bollate, Milan, Italy). All other chemicals, reagents, and water used in the study were of analytical grade. Experimental design and optimization were performed using Design-Expert software (13.0.0, Stat-Ease Inc., Minneapolis, MN, USA).

### 2.2. Initial Screening of Excipients

#### 2.2.1. Risk Assessment and Rationale for Excipient Selection 

In accordance with QbD and FbD principles, a preliminary risk assessment was conducted. Excipients were selected based on three criteria: technological functionality, regulatory acceptability, and potential intrinsic biological activity complementary to 7,8-DHF. ε-Polylysine, a naturally occurring homopolymer (MW ~4700 Da) produced by aerobic fermentation of *Streptomyces albulus* and recognized as GRAS by the FDA (GRN 000135), was selected as the cationic shell-forming polymer [21]. Its exposed positively charged amino groups provide the thermodynamic driving force for spontaneous polyelectrolyte complexation with the negatively charged phosphate domains of lecithin, forming a self-assembled lipid–polymer hybrid shell. Its biodegradation to L-lysine, a metabolically essential amino acid, ensures the absence of tissue accumulation or biological persistence [22]. Soybean lecithin, a plant-derived GRAS-listed phospholipid accepted in the FDA Inactive Ingredients Guide, was included as the interfacial stabilizer. Furthermore, the selection of soybean-derived lecithin reflects a preference for naturally sourced plant-based materials, in modest alignment with contemporary sustainability principles [23]. Soybeans are nitrogen-fixing crops that, through their symbiosis with Rhizobium bacteria, naturally reduce the demand for synthetic nitrogen fertilizers [24]. Beyond its structural role, lecithin serves as a biochemical precursor for choline, supporting endogenous acetylcholine synthesis and exhibiting intrinsic antioxidant activity [25,26]. Beyond its role in supporting cholinergic neurotransmission and cognitive function, lecithin has been reported to exhibit beneficial effects on lipid metabolism and metabolic disorders [27,28], potentially acting in synergism with 7,8-DHF in the context of neurometabolic disease. Fish oil was incorporated as a naturally derived FDA-approved liquid lipid excipient for intravenous use, providing structural integrity to the oily core [29]. Rich in long-chain omega-3 polyunsaturated fatty acids, notably DHA and EPA, fish oil contributes intrinsic antioxidant, anti-inflammatory, and neuroprotective properties, establishing a therapeutic complementarity with the encapsulated drug [30,31,32]. Similarly, fish oil represents a renewable marine-derived lipid whose extraction is increasingly performed via green technologies, such as supercritical CO_2_ or ultrasound-assisted extraction, reducing reliance on hazardous organic solvents traditionally employed in lipid processing [33,34,35]. The two surfactants investigated, Pluronic^®^ F127 and Tween^®^ 80, were selected for their well-established colloidal stabilization properties, biocompatibility, and documented safety profiles for nasal administration [36,37,38,39,40].

The preparation process employs ethanol and acetone as processing solvents, both classified as class 3 solvents according to ICH Q3C guidelines, indicating low toxic potential [41]. Both solvents are removed by evaporation during manufacturing.

Based on the literature’s evidence and preliminary formulation screening, drug amount, lecithin concentration, and surfactant type were identified as the variables most likely to influence CQAs and were therefore selected as factors for DoE investigation [42,43,44,45,46].

#### 2.2.2. Solubility Studies

Solubility of 7,8-DHF was determined in acetone and in fish oil via saturated solubility studies, as reported by Shah et al., with slight modifications [47]. Excess 7,8-DHF (10 mg/mL) was added into each screw-capped amber vial containing solvent or oil. The mixtures were continuously stirred for 48 h at 25 °C. After 48 h of stirring, equilibrium was achieved and 7,8-DHF dispersions were centrifuged at 10,000 rpm for 10 min at 25 °C. The concentration of 7,8-DHF in supernatant as assayed after appropriate dilution by UV spectroscopy (UH5300, Hitachi, Europe, Milan, Italy) at 328 nm. A 7,8-DHF calibration curve in acetone was used as reference (linear in the range 10–100 μg/mL, R^2^ = 0.998).

#### 2.2.3. Experimental Design

The traditional approach to developing a formulation is to change one variable at time. From this method, it is difficult to develop an optimized formulation, as the method cannot consider interactions between the variables. Hence, the combined influence of 3 independent variables in the preparation of 7,8-DHF nanoparticles (7,8-DHF NPs) was investigated via a 3-factor 2-level design; the numeric independent variables selected were concentration of 7,8-DHF (A) and concentration of soybean lecithin (B), and the categoric independent variable was surfactant type (C) as Pluronic^®^ F127 or Tween^®^ 80. Values of each variable are reported in Table 1. Experimental batches were performed for all eight possible combinations (Table 2). Mean particle size (Z-Ave), polydispersity index (PDI), zeta potential (ZP), and encapsulation efficiency (EE%) were inserted as dependent variables of the model and designated as factors Y_1_, Y_2_, Y_3_, and Y_4_, respectively. Statistical analysis was performed using Design-Expert software at a significance level of *p* < 0.05. The experimental conditions used to evaluate these variables are described in the Section 2.4 DLS Measurements, and Section 2.5 Encapsulation Efficiency.

### 2.3. Preparation of 7,8-DHF-Loaded Polylysine/Lecithin Nanoparticles

Nanoparticles (NPs) were prepared by a single-step procedure via solvent displacement method, as described by Alonso et al. [48]. Briefly, 7,8-DHF was first dissolved in acetone to obtain a clear drug solution. Lecithin was dissolved in 0.25 mL of warm ethanol (37 °C), and this solution was mixed with fish oil (0.7 mg/mL final concentration in the nanoparticle suspension) and the drug-containing acetone solution to a final organic phase volume of 5 mL. This organic phase was added dropwise over an external aqueous phase (1:2 ratio) containing surfactant (Pluronic^®^ F127 or Tween^®^ 80 0.1% *w*/*v*) in which polylysine (10 mg) was previously dissolved. Upon contact with the aqueous phase, rapid solvent diffusion drove nanoprecipitation, during which 7,8-DHF was physically entrapped within the forming matrix comprising lecithin, fish oil, and ε-polylysine, which act synergistically to maintain the drug in a physically stable, non-precipitated state. The resulting milky suspension was left under a fume hood to allow complete evaporation of organic solvents to a final volume of 10 mL.

### 2.4. DLS Measurements

Z-Ave, PDI, and ZP of NPs were determined by photon correlation spectroscopy and electrophoretic light scattering (NanoZS90; Malvern Instruments, Malvern, UK). The samples were diluted in water for ZP measurements (1:10) to obtain uniform dispersion with good scattering intensity. Three replicate measurements were recorded to ensure reproducibility.

### 2.5. Encapsulation Efficiency

To determine the encapsulation efficiency (EE%), NPs underwent centrifugation at 13,000 rpm for 80 min using a cooling centrifuge at 4 °C (Thermo Fischer Scientific Inc., Waltham, MA, USA). After the removal of supernatant, the pellet was dissolved in a mixture of ethanol/water 1:1 (V/V) and sonicated via a probe sonicator (Branson Sonifier 450, Marshall Scientific, Hampton, NY, USA), operating at 400 W power input for a total duration of 2 min at a constant frequency. The encapsulated 7,8-DHF concentration was determined from the 7,8-DHF calibration curve at 270 nm (λ max) via UV-VIS spectroscopy according to the absorbance of the dissolved pellets, using pellets from empty NPs as blanks, after proper dilution (1:10). The amount of drug entrapped was calculated according to Equation (1) [43,49].

(1) $$EE\%=\frac{amount\;of\;drug\;in\;the\;pellet}{total\;amount\;of\;drug}\times 100$$

The calibration curve in ethanol/water 1:1 (V/V) is linear in the range 0.25–20 μg/mL, with an R^2^ values of 0.998.

### 2.6. Optimization of 7,8-DHF NP Formulation

Desirability function and numerical optimization were applied to optimize the composition of the formulated 7,8-DHF NPs. The composition of the optimized formulation was determined using Design-Expert software after establishing the criteria for each response, as showcased in Table 3. The goal was to establish NPs with maximized ZP and EE%. The desirability function is a method for determining the best measurements for the independent factors; firstly, by assessing the desirability index for every dependent variable and, finally, to merge all responses in a single desirability function that ranges from 0 to 1 to represent the best values of the independent variables [50]. Triplicating the experiment facilitates checks of the accuracy and suitability of optimized conditions in the preparation of nanoparticles by controlling the formulation parameters. The optimized NP formulation was further characterized via TEM.

### 2.7. Transmission Electron Microscopy

Morphological examination was performed by means of transmission electron microscopy (TEM). Images were acquired using the Talos F200S G2 (ThermoFischer Scientific, Waltham, MA, USA) electron microscope in BF-STEM and HAADF mode. Samples were prepared by adding a drop of fresh particle suspension diluted at 0.1 mg/mL to a Ni mesh grid (Agar Scientific, Stansted, UK) and letting it dry in air. At least 4 images were acquired for each sample.

### 2.8. In Vitro Release Study

The in vitro drug release profile analysis of non-encapsulated 7,8-DHF (in PBS, pH 7.4:ethanol ratio 4:1) and encapsulated drug (7,8-DHF NPs) was performed using dialysis membranes with a MWCO of 3.5 kDa (standard grade regenerated cellulose membranes, SpectrumLabs, Los Angeles, CA, USA). Prior to use, the dialysis membranes were hydrated according to the manufacturer’s instructions. Specifically, a 2 mL volume of either the free drug solution (containing 500 μg of 7,8-DHF solubilized in PBS:ethanol 4:1 mixture) or the 7,8-DHF NPs suspension (at an equivalent drug concentration of 250 μg/mL, corresponding to an initial amount of 500 μg of 7,8-DHF) was loaded into the dialysis tubing. The drug solution and the NPs suspension were placed into dialysis tubing and incubated in 40 mL of the medium (PBS, pH 7.4:ethanol ratio 4:1), which was maintained under magnetic stirring at 37 °C. This system was employed to ensure sink conditions and adequate solubility of lipophilic 7,8-DHF throughout the release study, consistent with methodologies reported in the literature for poorly water-soluble compounds [51,52,53,54]. Release medium was sampled (2 mL) at predetermined time points (0, 30 min, 1, 2, 3, 4, 5, 6, 7, 8, 24, 48, 72 h) and immediately replaced with the same volume of medium, ensuring the volume of the external solution is constant. For each time point of the release study, the corresponding sample from unloaded NPs was used as a blank when analyzing the 7,8-DHF NPs to subtract background interference from the excipients. The absorbance was scanned at 318 nm, and the method showed linearity with a R^2^ of 0.999 in the range of 0.625–12.5 μg/mL. The percentage of 7,8-DHF released from the dialysis bag was calculated according to the following Equation (2):

(2) $$Cumulative\;drug\;release\left(\%\right)=\frac{C_{n}V+V_{i}\sum_{i=0}^{n-1}C_{i}}{\left[Total\;7,8-DHF\right]}\times 100$$

$C_{n}$: Concentration sampled for the nth time, μg/mL.

V: Total volume of released media, 40 mL.

$V_{i}$: Sampling volume at the ith point, 2 mL.

$C_{i}$: Sampling concentration at the ith point, µg/mL.

The release data were fitted to various mathematical models to elucidate whether the release mechanism followed, using zero-order, first-order, Higuchi, or Korsmeyer–Peppas models [55]. The best goodness-of-fit test (R^2^) was taken as a criterion for selecting the most appropriate model [56].

### 2.9. Stability Studies

Stability studies were carried out on the optimized 7,8-DHF NPs suspension according to International Conference on Harmonization (ICH) guidelines Q1A (R2) (stability testing) [57]. NPs were evaluated for long-term stability by keeping the samples at room temperature (RT), 40 ± 0.5 °C, and 75% ± 5% RH. Samples were withdrawn at 0, 30, 60, and 90 days, and Z-Ave, PDI and ZP were measured via DLS (NanoZS90, Malvern, UK).

### 2.10. DPPH Scavenging Activity

The DPPH scavenging assay was carried out as previously described with some modification [58]. Briefly, 100 µL samples (optimized 7,8-DHF NPs, blank NPs, and 7,8-DHF ethanolic solution) were mixed with 900 µL DPPH ethanolic solution (0.1 mM), vigorously shaken, and then incubated in the dark for 30 min at 25 °C. The solution absorbance was thus measured at 517 nm by UV-vis spectrophotometer (UH5300, Hitachi) using ethanol as a blank. The antioxidant activity of the samples was calculated by the following Equation (3):

DPPH scavenging activity (%) = (Ac − As)/Ac × 100(3)

where Ac is the absorbance of the control and As represents the absorbance of the test sample.

### 2.11. Stability in Simulated Fluids

To assess the stability and behavior of 7,8-DHF NPs formulation under physiological conditions, studies were carried out in simulated nasal fluid (SNF), artificial cerebrospinal fluid (aCSF), and SNF + 0.1% mucin. The SNF was prepared by dissolving 7.45 mg/mL NaCl, 1.29 mg/mL KCl, and 0.32 mg/mL CaCl_2_ · 2H_2_O in purified water and by adjusting the pH to 5.5 [59]. The composition of aCSF was as follows: NaCl 8.66 mg/mL; KCl 0.22 mg/mL; NaH_2_PO_4_ · 7H_2_O 0.03 mg/mL; MgCl_2_ · 6H_2_O 0.16 mg/mL; CaCl_2_ · 2H_2_O 0.21 mg/mL; and Na_2_HPO_4_ 0.03 mg/mL in purified water [60]. The NPs were incubated separately in each fluid at 37 °C under gentle agitation. Samples were collected at predefined time points to evaluate potential changes in size, PDI, and zeta potential. SNF and aCSF were prepared according to standard compositions reported in the literature, maintaining appropriate ionic strength and pH to mimic physiological environments.

### 2.12. In Vitro Assessments of NP–Mucin Interaction

The interaction between 7,8-DHF NPs and mucin was assessed using both ZP analysis and turbidimetric measurements. NPs were incubated with porcine gastric mucin (ratio 1:1) under physiological conditions. ZP was measured using a Zetasizer Nano ZS90 (Malvern Instruments) to evaluate surface charge variations as an indicator of interaction. Turbidity was assessed spectrophotometrically at 650 nm to detect possible aggregation or complex formation. Samples were diluted 1:10 with deionized water and analyzed at different time points: immediately after mixing (T0) and after 1 h and 24 h of incubation at 37 °C. All experiments were conducted in triplicate to ensure reproducibility.

### 2.13. Cell Cultures

OECs were selected as the cellular model for cytocompatibility studies based on their anatomical localization along the olfactory pathway and their biological relevance to intranasal nose-to-brain drug delivery. OECs are glial cells that surround olfactory axons along their entire route from the olfactory epithelium to the olfactory bulb, actively contributing to the maintenance and functional support of olfactory neurons [61]. Beyond their structural role, OECs play a relevant immunological function within the olfactory system, and are involved in the production of inflammatory mediators and protective factors in response to external stimuli or mucosal damage [62]. Previous studies have suggested that OEC-mediated immune responses may influence drug distribution and internalization following intranasal administration [62,63]. Considering these features, OECs represent a biologically relevant in vitro model for the preliminary evaluation of intranasally administered biopharmaceuticals [64]. In the present study, we compared the cytocompatibility profile of free 7,8-DHF and its nanoparticle formulation in this cellular model. As described by Chung et al., olfactory ensheathing cells (OECs) were isolated from the olfactory bulbs of 2-day-old mouse pups [65]. All procedures involving animals were conducted in compliance with Italian regulations on animal care (D.L. 116/92; 26/2014) and in accordance with the European Directive 2010/63/EU, with the goal of minimizing animal suffering and reducing the number of animals used. Neonatal mice were euthanized by decapitation, and their olfactory bulbs were excised and dissected in ice-cold Leibovitz’s L-15 medium (Sigma, Milan, Italy) supplemented with 25 mM HEPES. The tissue was then enzymatically digested for 15 min at 37 °C in Minimum Essential Medium-H (MEM-H) (Sigma, Milan, Italy) containing 0.03% collagenase and 0.25% trypsin (Sigma, Milan, Italy), following the protocol established by Pellitteri et al. (2007) [66]. Enzymatic digestion was halted by the addition of Dulbecco’s Modified Eagle Medium (DMEM) (Sigma, Milan, Italy) supplemented with 10% fetal bovine serum (FBS) (Sigma, Milan, Italy). The resulting cell suspension was mechanically dissociated using an 80 μm nylon mesh filter, followed by centrifugation at 500× *g* for 10 min. The cell pellet was then resuspended in fresh DMEM containing 10% FBS, 2 mM L-glutamine, 50 μg/mL penicillin, and 50 U/mL streptomycin (all from Sigma, Milan, Italy).

### 2.14. Treatment of OECs

OECs were plated in 96-well plates from Nunc™ MicroWell™ (Thermo Fisher Scientific) at a final density of 1 × 10^4^ cells/well. To obtain the desired concentrations for in vitro testing, the NPs formulation was concentrated by centrifugation at 13,000 rpm for 90 min at 4 °C. The supernatant was discarded, and the pellet was carefully resuspended in the appropriate volume of cell culture medium to reach the predetermined concentrations. The cells were treated with four concentrations (500 nM, 5 µM, 20 µM, 100 µM) of 7,8-DHF, comparing the encapsulated one with the free drug. Finally, the treated cells were incubated at 37 °C in a humidified chamber (5% CO_2_–95% air) for 24 h. Untreated cells were used as a control.

### 2.15. Cellular Viability

Cell viability of OECs was assessed using a quantitative colorimetric assay based on the reduction of 3-(4,5-dimethylthiazol-2-yl)-2,5-diphenyltetrazolium bromide (MTT) [67]. Briefly, MTT was added to each well at a final concentration of 1.0 mg/mL. After 2 h of incubation in a CO_2_ incubator, the culture medium was carefully removed, and an acid-isopropanol/SDS solution (used as MTT solvent) was added to halt the enzymatic conversion of MTT (yellow) into formazan (purple). Subsequently, the plates were placed on an orbital shaker for 15 min to ensure complete solubilization of the formazan crystals. Absorbance was then measured at 570 nm using a multi-well plate reader (Multiskan, Flow Laboratories, Helsinki, Finland). Results are expressed as the percentage of MTT reduction relative to untreated control cells. Cell viability was calculated using the following Equation (4):

(4) $$Cell\;viability\left(\%\right)=\frac{{Int}_{s}}{{Int}_{control}}\times 100$$

where Int_s_ is the colorimetric intensity of cells incubated with NPs, and Int_control_ is the colorimetric intensity of untreated cells.

### 2.16. Statistical Analysis

Statistical analysis was performed using Prism 6 (GraphPad Software, San Diego, CA, USA). Data were analyzed using one-way or two-way ANOVAs, followed by the appropriate post hoc test (Holm–Sidak’s or Tukey’s) for multiple comparisons, depending on the experimental design. A *p*-value < 0.05 was considered statistically significant.

## 3. Results and Discussions

### 3.1. The 7,8-DHF Solubility Test

Preliminary solubility studies were conducted to evaluate the suitability of the selected solvent and oily phase for the intended formulation strategy. Since no data are currently available in the literature regarding the solubility of 7,8-DHF in acetone, a solubility study was carried out to assess its suitability for use in the formulation process. Previous reports indicate a solubility of ~2 mg/mL in ethanol and up to 50 mg/mL in DMSO, but no specific information exists for acetone. The results obtained in this study revealed a solubility of approximately 4.8 mg/mL in acetone, indicating that this solvent can be effectively used for drug incorporation in the nanoencapsulation process.

Regarding 7,8-DHF solubility in purified fish oil, the results indicated approximately 2.025 μg/mL. Such values are considered adequate to prepare the NP suspensions in the experimental conditions of this study [68].

### 3.2. Relative Impact of Independent Variables on the Responses

To rationally guide the formulation process and identify the most influential variables affecting nanocarrier properties, a DoE approach was applied. This statistical method enabled a systematic and efficient exploration of multiple formulation parameters simultaneously, reducing the number of experimental runs while maximizing information output (Table 4). Through the screening and optimization phases, DoE allowed the identification of optimal conditions to obtain nanocarriers with the desired physicochemical characteristics, such as Z-Ave (Y_1_), PDI (Y_2_), ZP (Y_3_), and EE% (Y_4_). The statistical analysis of the obtained results by 2^3^ full factorial design was performed by ANOVA. Although most effects were not statistically significant at the conventional thresholds (t-value or Bonferroni limits), the polynomial models were built considering the most influential terms, as suggested by the Pareto charts, following a hierarchical model structure to explore potential trends and interactions [69]. DoE enables the analysis of both the qualitative and quantitative effect of variables on response parameters. Here, the positive sign (+) in polynomial equations indicates that an increase in the level of one variable causes an increase in the corresponding response parameter, while the negative sign (−) indicates that an increase in the level of one variable causes a decrease in the response parameter (Equations (5)–(8)).

**Figure 1:**
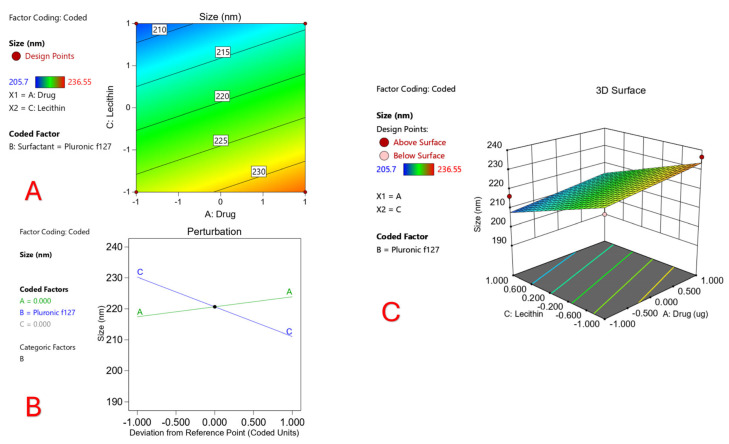
Model graphs: (**A**) contour plot; (**B**) perturbation chart; (**C**) three-dimensional plot of particle size (where categoric factor B is Pluronic^®^ F127).

**Figure 2:**
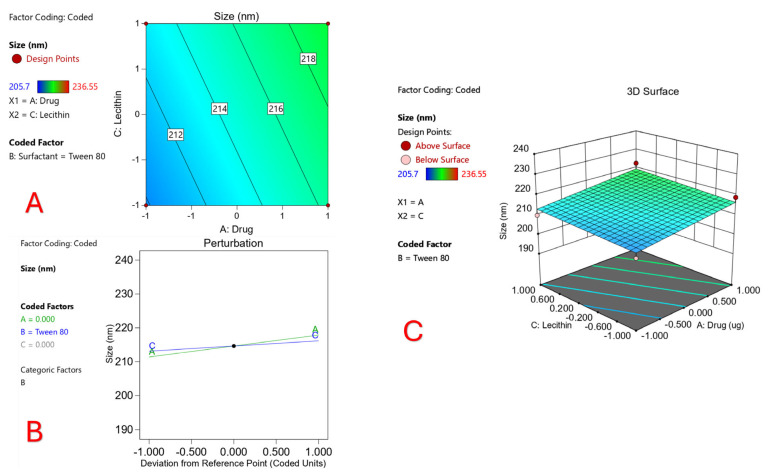
Model graphs: (**A**) contour plot; (**B**) perturbation chart; (**C**) three-dimensional plot of particle size (where categoric factor B is Tween^®^ 80).

**Figure 3:**
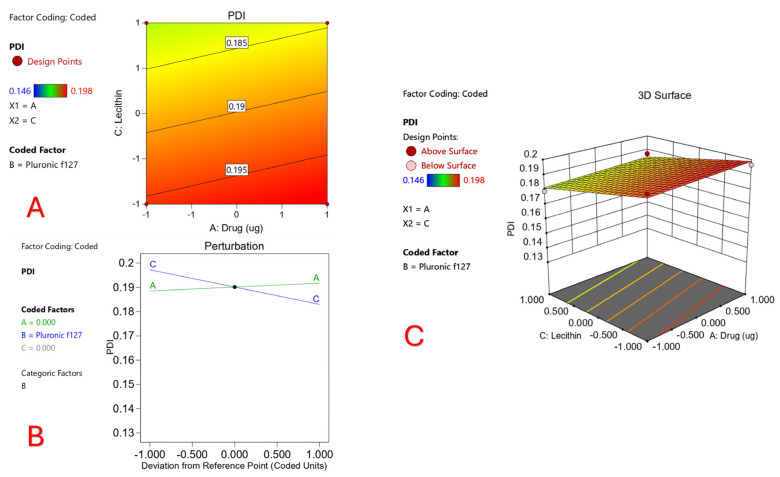
Model graphs: (**A**) contour plot; (**B**) perturbation chart; (**C**) three-dimensional plot of PDI (where categoric factor B is Pluronic^®^ F127).

**Figure 4:**
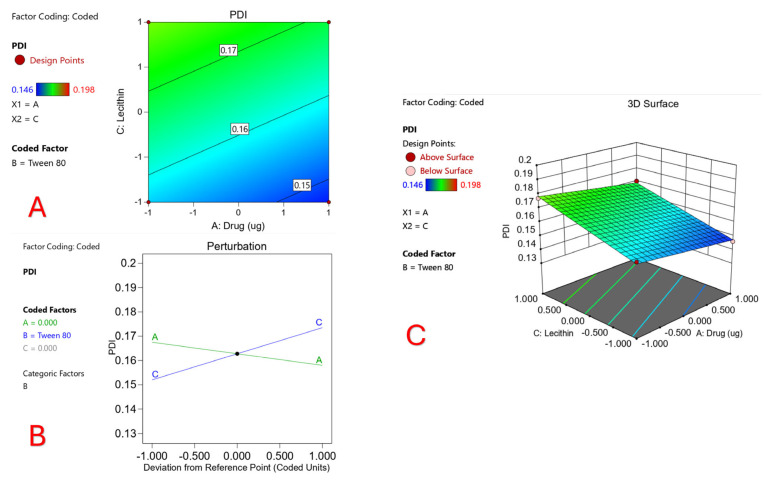
Model graphs: (**A**) contour plot; (**B**) perturbation chart; (**C**) three-dimensional plot of PDI (where categoric factor B is Tween^®^ 80).

**Figure 5:**
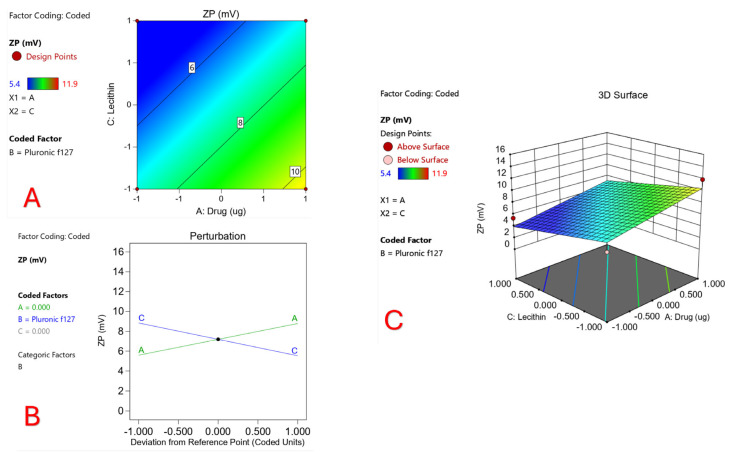
Model graphs: (**A**) contour plot; (**B**) perturbation chart; (**C**) three-dimensional plot of ZP (where categoric factor B is Pluronic^®^ F127).

**Figure 6:**
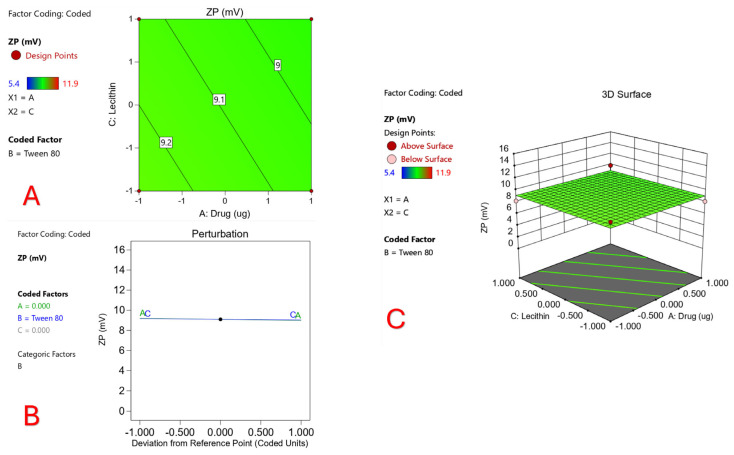
Model graphs: (**A**) contour plot; (**B**) perturbation chart; (**C**) three-dimensional plot of ZP (where categoric factor B is Tween^®^ 80).

**Figure 7:**
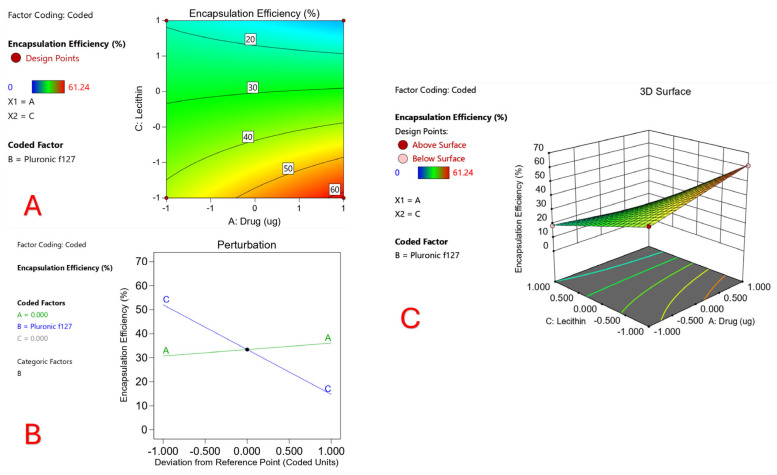
Model graphs: (**A**) contour plot; (**B**) perturbation chart; (**C**) three-dimensional plot of EE% (where categoric factor B is Pluronic^®^ F127).

**Figure 8:**
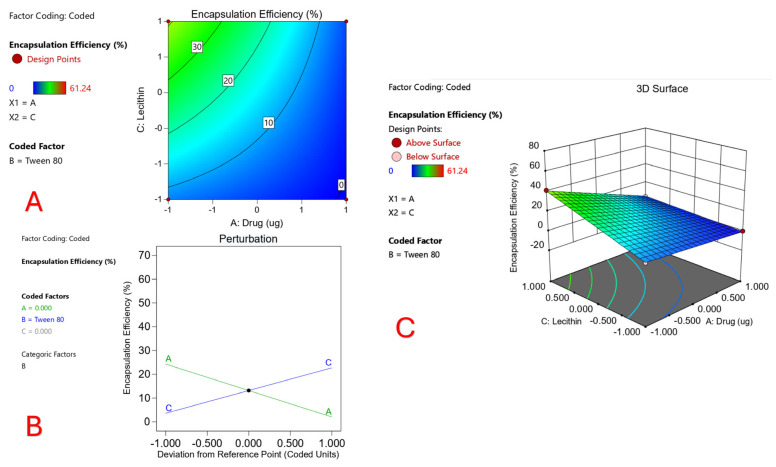
Model graphs: (**A**) contour plot; (**B**) perturbation chart; (**C**) three-dimensional plot of EE% (where categoric factor B is Tween^®^ 80).

Intending to visualize the relationship between responses and formulation variables, model graphs, namely perturbation charts, contour plots, and 3D response surfaces, were generated to assess the individual and interactive effects on the response (Figure 1, Figure 2, Figure 3, Figure 4, Figure 5, Figure 6, Figure 7 and Figure 8; for each A, B, and C).

#### 3.2.1. Effect of Independent Variables on NP Size

The effects of the varying three factors (drug amount, lecithin amount, and surfactant type) on the mean particle size of 7,8-DHF NPs are shown in Figure 1 and Figure 2. The particle size obtained at the low (−) and high (+) levels of the investigated factors ranged from 205.7 to 236.55 nm across the eight experimental batches.

The experimental data were analyzed using Design-Expert^®^ software and fitted to a two-factor interaction (2FI) model. Significant effects were identified through half-normal plot and Pareto chart analyses. The Pareto chart highlighted that the interaction between surfactant type and lecithin concentration (BC) exerted the greatest influence on particle size, with a percentage contribution of 33.83%. This was followed by lecithin concentration (C), drug amount (A), and surfactant type (B), contributing 17.84%, 11.48%, and 9.94%, respectively.

A reduced 2FI model including A, B, C, and BC terms was generated, and the resulting empirical equation is reported below (5):

Size = + 217.4 + 3.24 × A + 3.02 × B − 4.04 × C − 5.57 × BC(5)

ANOVA indicated that the overall 2FI model was not statistically significant (F = 2.04, *p* > 0.1000), likely due to the limited experimental degrees of freedom associated with the unreplicated 2^3^ full factorial design. Consequently, the resulting empirical equation (Equation (5)) and the Pareto chart should be interpreted solely as a descriptive visualization of observed trends rather than a predictive statistical model.

Descriptive analysis of the experimental data suggests that shifting from Pluronic^®^ F127 to Tween^®^ 80 resulted in a slight reduction in the particle size (Figure 1 and Figure 2). Using Pluronic^®^ F127 resulted in larger particle sizes, likely due to its high molecular weight (12,000 Da) and the formation of a thick steric hydration layer at the particle interface. In contrast, as observed in another study on the optimization of polymeric NPs, Tween^®^ 80 led to smaller particles, attributable to its lower molecular weight (1.310 Da), superior interfacial adsorption efficiency, and enhanced reduction in interfacial tension, facilitating finer emulsification.

The role of lecithin in the formation and stabilization of NPs appeared to be closely related to the type of surfactant used. Although its main purpose was to promote electrostatic interactions with ε-polylysine within the NP shell, lecithin also exhibited intrinsic surface-active properties that significantly influenced particle size in combination with Pluronic^®^ F127. Despite the constant amount of Pluronic^®^ F127 in all formulations, lower concentrations of lecithin resulted in larger particles. Conversely, increasing lecithin led to smaller particles, suggesting a synergistic interaction between lecithin and interfacial film formed by Pluronic^®^ F127 (Figure 1B).

When Tween^®^ 80 was used as the primary surfactant, variations in lecithin concentration did not significantly affect the particle size and led to smaller particles, as was observed in another study (Figure 2B) [70]. This may be explained by the dense, compact interfacial film formed by Tween^®^ 80, possessing a large, strongly hydrophilic ethoxylated sorbitan headgroup, which likely hindered the incorporation or functional contribution of lecithin at the interface [71].

The non-significance of the overall 2FI model is attributable to the limited statistical power inherent to an unreplicated 2^3^ full factorial design, which provides very few degrees of freedom for error estimation. Additionally, the experimental domain was deliberately constrained based on preliminary formulation screening studies conducted with blank nanoparticles, in which formulations prepared at ε-polylysine:lecithin ratios of 1:1 and 1:2 generated nanoparticles with mean diameters of approximately 200 nm and low PDI values, whereas increasing the ratio to 2:1 resulted in substantially higher polydispersity. The investigated lecithin range may therefore not have been sufficiently broad to produce statistically detectable effects on particle size. It should be noted, however, that preformulative screening was conducted in the absence of 7,8-DHF; the DoE was subsequently used to investigate whether the inclusion of this molecule would introduce additional variability in colloidal properties. Within the investigated region, particle size remained relatively constant across all experimental runs (205.7–236.5 nm), indicating that all tested compositions could produce colloidally stable nanoparticles. Given the non-significance of the overall model, the following observations are offered as descriptive interpretations of the observed trends, consistent with the physicochemical properties of the formulation components, and should not be construed as statistically supported conclusions.

#### 3.2.2. Effect of Independent Variables on NP PDI

The polydispersity index (PDI) is a parameter used to evaluate the homogeneity of nanoparticle formulations. The tested formulations exhibited PDI values ranging from 0.146 to 0.198, indicating a narrow and homogeneous particle size distribution (Figure 3 and Figure 4).

The experimental data were analyzed using Design-Expert^®^ software and fitted to a two-factor interaction (2FI) model. Pareto chart analysis indicated that the surfactant type (B) and the interaction between surfactant type and lecithin concentration (BC) exerted the greatest influence on PDI, with percentage contributions of 65.34% and 27.86%, respectively. These were followed by the interaction between drug amount and surfactant type (AB) and lecithin concentration (C), contributing 3.54% and 1.15%, respectively.

A reduced 2FI model including A, B, C, AB, and BC terms was generated, and the resulting empirical equation is reported below (6):

PDI = + 0.1764 − 0.0016 × A + 0.0137 × B + 0.0018 × C + 0.0032 × AB − 0.0089 × BC(6)

The high F-value of 31.44 and the low *p*-value of 0.0311 indicate that the model is statistically significant, with an adequate goodness of fit.

The statistical analysis showed that the PDI was significantly influenced by the type of surfactant used, as well as by its interaction with lecithin, though the primary effect was attributable to the surfactant itself. Consistent with our previous observations on particle size, different surfactants exert distinct effects on the NP characteristics (Figure 3 and Figure 4). Pluronic^®^ F127 and Tween^®^ 80 differ markedly in their molecular structure and interfacial behavior; Pluronic^®^ tends to produce larger particles, likely due to its ability to interact with other components and form less compact interfacial layers, whereas Tween^®^ 80 forms a tightly packed interfacial film that favors smaller and more uniform particles. While lecithin can modulate the interface, its influence on PDI appears secondary compared to the dominant effect of the surfactant type.

#### 3.2.3. Effect of Independent Variables on NP ZP

The effects of the varying three factors (drug amount, lecithin amount, surfactant type) are demonstrated in Figure 5 and Figure 6. The experimental data were analyzed using Design-Expert^®^ software and fitted into a two-factor interaction (2FI) model. The results indicated that the interaction between the three independent variables (ABC) exhibited the highest effect in altering ZP, with a percentage contribution of 28.34%. The effect of each independent variable and AB and BC combinations showed similar percentage contributions—drug concentration (A) 10.97%, surfactant type (B) 17.93%, and lecithin concentration (C) 14.61%, while AB and BC combinations were 14.36% and 12.41%, respectively. To obtain a hierarchical model, the ABC term was excluded from the equation and terms with higher contributions were selected, producing the following equation (Equation (7)):

ZP = + 8.14 + 0.7450 × A − 0.9525 × B + 0.8600 × C + 0.8525 × AB − 0.7925 × BC(7)

ANOVA indicated that the overall 2FI model was not statistically significant (F = 0.95, *p* > 0.1000), which may be attributed to the limited degrees of freedom associated with the unreplicated 2^3^ full factorial design. ZP values appeared influenced by the type of surfactant and, in the case of Pluronic^®^ F127-based formulations, also by the amount of lecithin and drug (Figure 5). When Tween^®^ 80 was used as the surfactant, ZP remained relatively constant across all formulations, suggesting the formation of a compact and stable interfacial film that masks potential surface charge variations (Figure 6) [72]. Conversely, in Pluronic^®^-based formulations, ZP became more positive with decreasing lecithin concentrations and increasing drug content.

Although polylysine was included, carrying positively charged amino groups, the ZP of formulations was not strongly positive. This is attributable to partial masking of the polylysine residues by the negatively charged DHA moieties from the fish oil, which interact at the nanoparticle surface. A similar behavior has been reported for nanocapsules containing oleic acid, where the fatty acid’s negative charge significantly reduced the net surface potential despite the presence of positively charged residues [73]. Thus, the observed ZP values do not indicate insufficient polylysine incorporation; instead, they reflect a natural modulation of surface charge due to interactions with DHA and lecithin, which nonetheless contribute to colloidal stability. Furthermore, it should be noted that ε-polylysine, the primary determinant of surface charge in this system due to its polycationic nature, was maintained at a fixed amount (10 mg) across all experimental runs. This may have further constrained the variability in ZP values, as the dominant contributor to surface charge was not varied within the DoE. The observed differences in ZP across formulations therefore reflect the modulating effect of lecithin, surfactant type, and drug amount on the baseline charge established by the fixed ε-polylysine content rather than the independent contributions of each factor to surface charge per se. Regarding the interpretation of coefficient signs for both responses and given the non-significance of the overall model, the following observations are offered as descriptive interpretations of the observed trends, consistent with the physicochemical properties of the formulation components, and should not be construed as statistically supported conclusions.

#### 3.2.4. Effect of Independent Variables on NP EE%

The effectiveness of 7,8-DHF entrapment within the formulations was evaluated to investigate the impact of NP composition on EE% (Figure 7 and Figure 8). The EE% values obtained for the eight experimental batches ranged from 0 to 61.24%.

The experimental data were analyzed using Design-Expert^®^ software and fitted to a two-factor interaction (2FI) model. Analysis of the half-normal and Pareto charts indicated that the interaction between surfactant type and lecithin concentration (BC) exhibited the highest effect on EE%, with a percentage contribution of 45.49%. This was followed by surfactant type (B) (23.46%), the interaction between drug amount and lecithin concentration (AC) (11.28%), and the interaction between drug amount and surfactant type (AB) (10.90%).

To maintain model hierarchy, the three-factor interaction term (ABC) was excluded, and a reduced 2FI model including A, B, C, AB, AC, and BC terms was generated.

The resulting model equation is reported below (8):

EE% = + 23.29 − 4.23 × A + 10.13 × B − 4.54 × C + 6.91 × AB − 7.02 × AC − 14.11 × BC(8)

ANOVA indicated that the overall 2FI model was statistically significant (F = 269.42, *p* = 0.0466). The significant model terms were B, AB, AC, and BC (*p* < 0.05). EE% was primarily influenced by the interaction between surfactant and lecithin (BC), followed by the type of surfactant (B) and, to a lesser extent, the interaction between drug and lecithin concentrations (AC). This underscores the central role of interfacial composition and compatibility in determining the drug-loading capacity of NPs. The surfactant–lecithin interaction likely modulates the structure and stability of the interfacial film, enhancing or reducing the retention of lipophilic drugs within the oil core.

Interestingly, higher EE% values were observed when Pluronic^®^ F127 was used as the surfactant compared to Tween^®^ 80. This may be attributed to the unique steric stabilization properties of Pluronic^®^, which, due to its PEO-PPO-PEO block copolymer structure, can create a more stable and structured interfacial layer. Such organization may limit drug diffusion from the core and enhance encapsulation, as supported by studies reporting the efficient use of Pluronic^®^-based nanocarriers for hydrophobic drugs [74].

The interaction between drug and lecithin concentrations revealed that higher drug content and lower lecithin amounts were associated with increased EE%. This may be due to enhanced solubilization and partitioning of the lipophilic drug into the oily core at higher concentrations, while excessive lecithin could destabilize the interfacial film or compete for space, thus hindering drug retention. These findings are in line with previous studies comparing the interaction between lecithin amount and EE% of lipophilic compounds [75].

The EE% of 0% observed for STD2 (Tween^®^ 80, 500 μg drug, 5 mg lecithin) was not an isolated anomaly but rather the predictable outcome of the simultaneous convergence of all compositional factors unfavorable to drug encapsulation, as supported by the statistically significant 2FI model (F = 269.42, *p* = 0.0466). Specifically, the ANOVA identified the BC interaction (surfactant type × lecithin concentration) as the dominant factor affecting EE% (45.49% contribution, *p* < 0.05), with a strongly negative coefficient (−14.11) when Tween^®^ 80 is combined with low lecithin concentration. This indicates that Tween^®^ 80, unlike Pluronic^®^ F127, does not form a sufficiently structured interfacial film to retain the lipophilic drug, an effect that is amplified at low lecithin concentrations, where the interfacial film is least reinforced. Additionally, the AC interaction (drug amount × lecithin concentration, coefficient −7.02, *p* < 0.05) further penalizes the combination of high drug load with low lecithin, likely due to saturation of the interfacial retention capacity relative to the available phospholipid. STD2 therefore represents the extreme case in which all three unfavorable conditions—Tween^®^ 80 as a surfactant, a minimum lecithin concentration, and a maximum drug load—act simultaneously and synergistically to prevent drug retention within the nanoparticle core.

### 3.3. Optimization

Based on the statistical model generated through DoE, an optimization study was carried out to define the most favorable combination of formulation parameters. The optimization process relied on the interpretation of the response surfaces and desirability profiles, enabling the identification of the critical factor levels required to achieve optimal response values. The strategy was designed to ensure robust performance of the formulation within the defined design space, in compliance with QbD principles [76,77].

**Figure 9:**
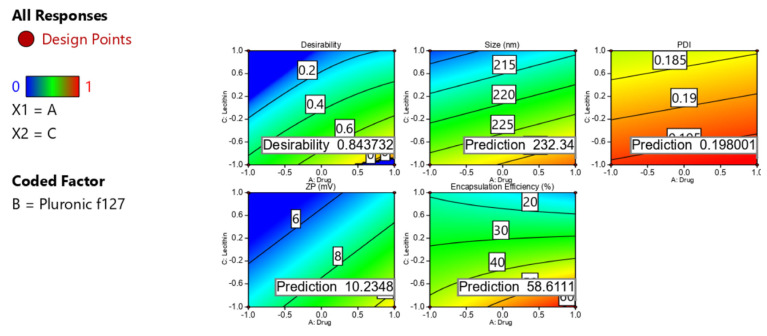
Graphical representation of contour plots showing the effect of variables on particle size, PDI, ZP, and EE% through a desirability tool.

Regression model developed in this study was used to identify out the optimal conditions to prepare NPs (Figure 9). Given that particle size and PDI values were already acceptable across all experimental conditions, these parameters were assigned an “in range” objective during the optimization to prioritize the fine-tuning of more critical variables, such as ZP and EE%. An algebraic solution for preparing the desired NPs was presented using software in the coded form and was found to be A = 0.999; B = Pluronic^®^ F127; and C = −8.777 (actual drug = 500 µg; surfactant = Pluronic^®^ F127; lecithin = 5.30), with a desirability value of 0.811. Under these optimal conditions, the predicted particle and experimental values of quadruplicate experiments are reported in Table 5. Predictions for particle size and PDI showed good agreement with the experimental data, with relative errors below 6%, confirming the model’s reliability for these parameters. ZP exhibited a larger discrepancy (relative error 42%), suggesting that predictions for this parameter are less precise, likely due to non-linear effects or experimental factors not included in the model. The encapsulation efficiency (EE%) was predicted slightly higher than the experimental value (relative error ~10%) but in line with the intended formulation goal, indicating that the model can also be useful for estimating desired optimal values. Moreover, 24.0% of free drug recovered in the supernatant demonstrates a robust and reproducible total drug recovery (mass balance) of 89.0% (n = 3).

### 3.4. Comprehensive Characterization of the Optimized Formulation

#### 3.4.1. TEM Images

**Figure 10:**
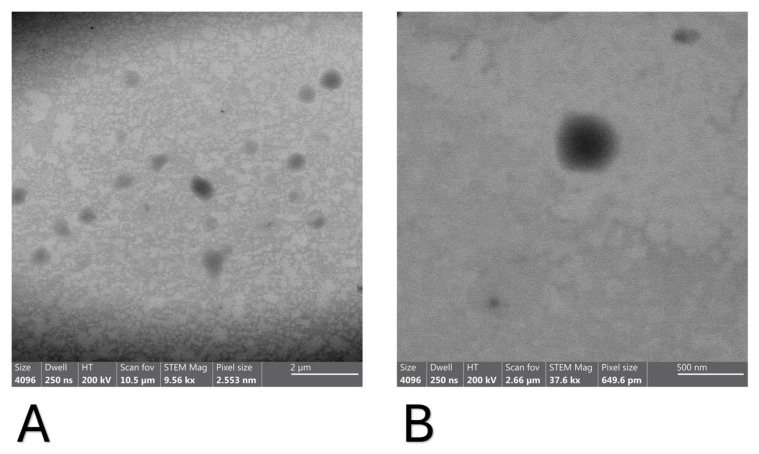
Morphological analysis of NPs illustrating a wide field (**A**) or detail of a single NP (**B**).

Transmission electron microscopy (TEM) images show the presence of spherical nanoparticles in the nanometric range (below 500 nm) with good homogeneity, confirming DLS data (Figure 10).

The low electron density of the matrix and the apparent blurring of objects are consistent with the presence of oil in the composition, confirming the successful formation of the nanosystem.

#### 3.4.2. In Vitro Release Profiles

**Figure 11:**
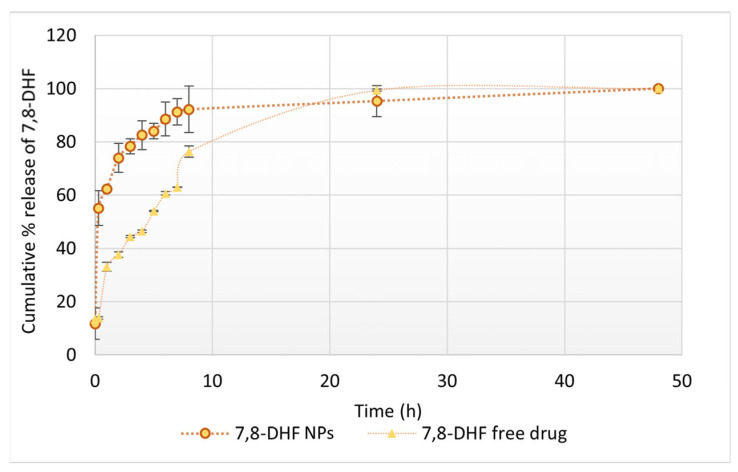
Cumulative percent 7,8-DHF dissolution or released from NPs over a period of 48 h.

The release profiles of 7,8-DHF as free drug or from NP formulations are displayed in Figure 11.

The release kinetics was evaluated by fitting the data into first-order, zero-order, Higuchi, and Korsmeyer–Peppas models (Table 6).

The release kinetics analysis showed that 7,8-DHF-loaded NPs best fit the Higuchi model (R^2^ = 0.9937). This high correlation coefficient indicates that, despite a prominent initial burst effect (~60% within the first hour) driven by the drug fraction localized near the nanoparticle surface, the subsequent progressive release follows a matrix-diffusion mechanism governed by Fick’s law. Such biphasic release profiles, characterized by an immediate burst phase followed by a sustained, diffusion-driven Higuchi pattern, were documented for polymeric and lipid nanocarriers, where the initial rapid release does not preclude a good mathematical fit for the subsequent matrix-controlled diffusion [78].

The lower correlation coefficients observed for the NP formulation with zero-order (R^2^ = 0.2778) and first-order (R^2^ = 0.6625) further confirm that drug release from NPs is predominantly governed by a diffusion-based mechanism through the lipid–polymer matrix. The low correlation coefficient for Korsmeyer–Peppas model (R^2^ = 0.3463) is mathematically expected, as this specific model is strictly valid only for the first 60% of cumulative drug release, a threshold immediately reached and surpassed by our system during the initial burst phase and previously observed for similar NP systems [78].

The free drug followed first-order kinetics (R^2^ = 0.8099). Conversely, the free drug exhibited poorer fitting to zero-order (R^2^ = 0.655), Higuchi (R^2^ = 0.6768), and Korsmeyer–Peppas (R^2^ = 0.6145) models, supporting a concentration-dependent free diffusion across the dialysis membrane.

It should be noted that the nanoformulation achieved complete drug release within 48 h, and the overall release profiles do not indicate sustained or prolonged release in the pharmacokinetic sense.

#### 3.4.3. ROS Scavenging Capacity

As 7,8-DHF possesses well-established antioxidant properties due to its flavone backbone (C6–C3–C6) and the presence of hydroxyl groups, this enables hydrogen atom donation and electron transfer reactions, which are essential for radical scavenging activity in the DPPH assay [79].

To investigate the contribution of individual formulation components to the observed radical scavenging capacity, the DPPH activity of blank NPs, individual excipients, and a physical mixture of all components with free 7,8-DHF at equivalent concentrations, was assessed. Individual excipients, such as lecithin, ε-polylysine, Pluronic^®^ F127, and fish oil, exhibited negligible to modest radical scavenging activity (2–11%). Blank NPs showed a scavenging capacity of approximately 20% at T0, reflecting the cumulative contribution of the excipient matrix in the absence of drug (Appendix A, Figure A2).

**Figure 12:**
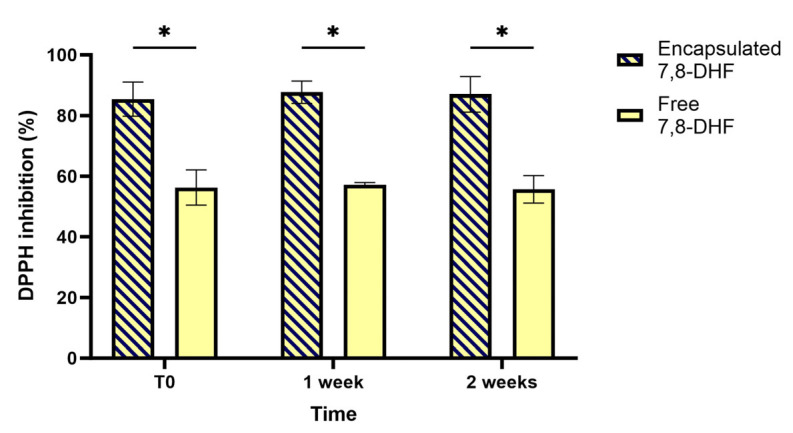
Time-dependent antioxidant activity of free and encapsulated 7,8-dihydroxyflavone (7,8-DHF) assessed by DPPH assay. Data are expressed as mean ± SEM. Two-way repeated-measures ANOVA, with time as the within-subject factor and encapsulation status (free vs. encapsulated) as the between-condition factor, revealed a significant effect of time (*p* = 0.0020) and encapsulation status (*p* = 0.0021) but no significant time × encapsulation status interaction (*p* = 0.4293). Post hoc paired comparisons were performed using the Holm–Šídák method to compare free and encapsulated 7,8-DHF at each time point. * *p* < 0.05 versus free 7,8-DHF at the corresponding time point.

DPPH radical scavenging capacity was higher for 7,8-DHF encapsulated in NPs compared to its free form, both at T0 and throughout the storage period (Figure 12). At T0, the encapsulated formulation showed an inhibition percentage exceeding 80%, whereas the free drug displayed a lower activity close to 58%. This difference is largely explained by the additive contribution of the excipient matrix (~20%, as shown by blank NPs) rather than by any intrinsic enhancement of 7,8-DHF radical scavenging activity upon encapsulation.

The decline observed in the free drug can also be attributed to chemical instability in the medium, including oxidation, or structural rearrangements that are known to affect flavonoid-like molecules [80].

Moreover, the NP formulation not only slowed this degradation but also appeared to maintain or even slightly enhance antioxidant performance during the first weeks of storage. The presence of soybean lecithin in the formulation may contribute to this enhanced antioxidant performance. As reported in lecithin-containing SNEDDS, lecithin stabilizes bioactive compounds and improves radical scavenging efficiency by creating a protective environment that mitigates oxidative degradation [81]. Therefore, the combined effects of excipient synergy and lecithin-mediated stabilization appear to preserve the radical scavenging capacity of 7,8-DHF.

#### 3.4.4. In Storage Stability

The stability of 7,8-DHF NPs was evaluated for 90 days in terms of the physicochemical characterization (Z-Ave, PDI, and ZP) of the formulations stored at RT and 40 ± 0.5 °C and 75 ± 5% RH, according to ICH guidelines Q1A (R2) (stability testing).

**Figure A1:**
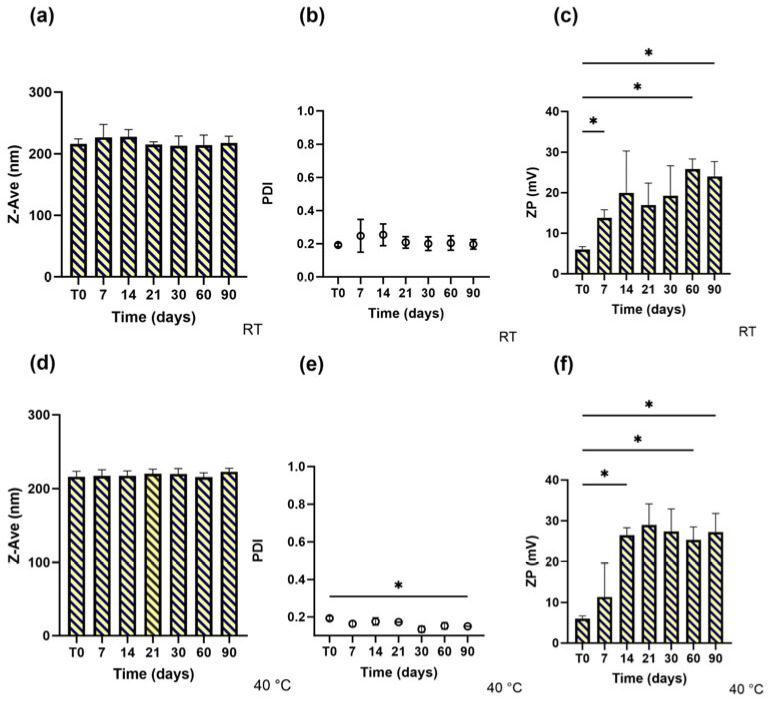
Size (**a**,**d**), PDI (**b**,**e**), and ZP (**c**,**f**) stability of NPs stored at room temperature (RT) and at 40 °C for 90 days. Data are presented as mean ± standard deviation (*n* = 3). Differences across the storage time points were analyzed using one-way repeated measures ANOVA followed by multiple comparison testing, * *p* < 0.05.

**Figure A2:**
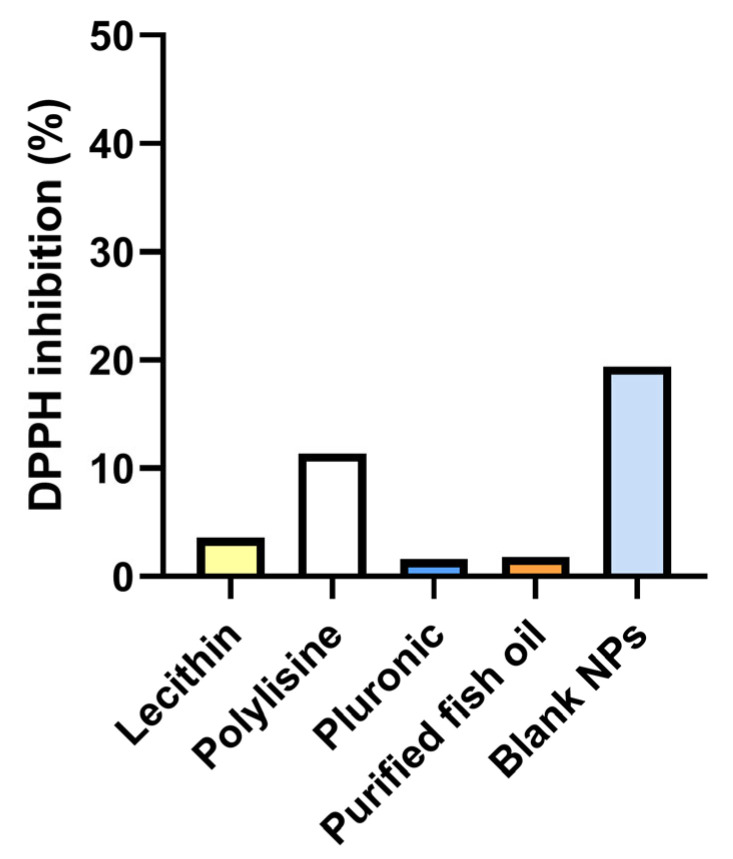
DPPH radical scavenging capacity (% inhibition) of individual formulation excipients (lecithin, ε-polylysine, Pluronic^®^ F127, purified fish oil)*,* tested at concentrations equivalent to those present in the NP formulation and blank NPs.

The particle size remained constant throughout the study at both storage conditions, with no significant increase compared to the initial values. Similarly, the PDI values were consistently below 0.3, confirming the homogeneity of the colloidal dispersion and the absence of significant aggregation phenomena (Appendix A, Figure A1).

In contrast, significant changes were observed in ZP values. While the initial ZP was relatively low (~+5 mV), it progressively increased over time, reaching values between +20 and +30 mV, with fluctuations particularly evident at later time points (Appendix A, Figure A1). The observed trend suggests that the surface properties of NPs are not completely stable over time; therefore, storage in lyophilized form with reconstitution prior to use is recommended.

#### 3.4.5. NP Stability in Simulated Fluids

The colloidal stability of NPs in simulated biological fluids is a critical parameter for their potential application in intranasal delivery and subsequent brain targeting. Physiological environments that are rich in salts, proteins, and mucopolysaccharides can trigger aggregation or destabilization phenomena and compromise drug delivery performance [82].

**Figure 13:**
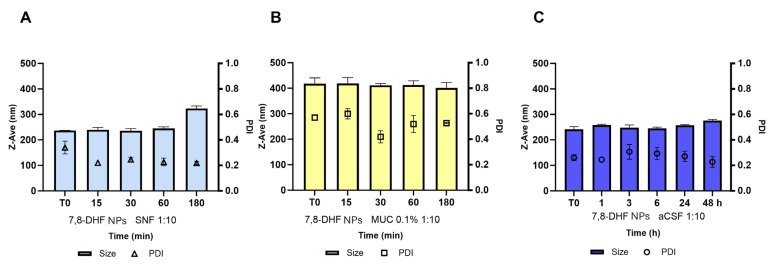
Stability studies (changes in mean size and PDI) of 7,8-DHF NPs incubated in (**A**) SNF, (**B**) SNF with 0.1% (*w*/*v*) mucin, and (**C**) aCSF at 37 ± 0.5 °C over time. The ratio between formulation and simulated fluid is 1:10. Values represent the mean of three technical replicates on the same sample, reflecting measurement precision rather than sample-to-sample variability.

To study the behavior of NPs after intranasal administration and the influence of ions present in SNF, mucin (MUC), and aCSF, these were incubated in SNF, SNF with 0.1% (*w*/*v*) of MUC, and aCSF and at 37 ± 0.5 °C. The incubation of 7,8-DHF NPs in SNF (Figure 13A) demonstrated good colloidal stability over time. Z-Ave remained in the range of ~220–250 nm across the entire incubation period (up to 180 min). Polydispersity index (PDI) values were consistently below 0.3, indicating a narrow size distribution and homogeneous dispersion.

The addition of mucin (0.1% *w*/*v*) to SNF did not affect the stability of the NPs (Figure 13B). Particle size values remained between ~400–450 nm during incubation, while PDI remained below 0.4 for up to 180 min, suggesting only minor interactions with mucin components.

Incubation in aCSF (Figure 13C) confirmed the stability of 7,8-DHF NPs for up to 48 h. Hydrodynamic diameters were maintained in the range of 220–250 nm, and PDI values remained consistently low (<0.3), demonstrating colloidal stability, even in a medium mimicking cerebrospinal condition.

In our experiments, 7,8-DHF NPs preserved their size and dispersity in both nasal and cerebrospinal fluid simulations, which agrees with previous reports on polymeric nanoparticles that demonstrated robust colloidal stability under similar conditions [83].

The interaction with mucin is relevant for intranasal administration. The literature’s evidence indicates that nanoparticles often experience size enlargement in mucin-rich environments due to adsorption and complex formation [84]. The results here show a moderate increase in particle size in the presence of MUC but without significant destabilization, suggesting a surface-level interaction insufficient to induce aggregation. This observation is consistent with studies reporting that nanoparticles designed for controlled mucoadhesion often display moderate size increases in mucin but maintain colloidal stability [84]. Such behavior can be advantageous for nasal retention.

It should be noted that the mucin concentration employed (0.1% *w*/*v*) was selected to be consistent with established in vitro screening methodologies for DLS-based NP–mucin interaction studies, where sub-physiological concentrations provide a sensitive and reproducible assessment without the optical interference associated with high-concentration mucin gels. While this approach does not fully replicate the viscoelastic properties of native nasal mucus, it represents a validated preliminary tool for evaluating colloidal behavior in mucin-containing media [84,85,86,87].

The long-term stability in aCSF (up to 48 h) is noteworthy. The maintenance of hydrodynamic diameters below 300 nm and low PDI values suggests that the NPs resist precipitation or aggregation in cerebrospinal-like conditions.

#### 3.4.6. Mucin Interaction

**Figure 14:**
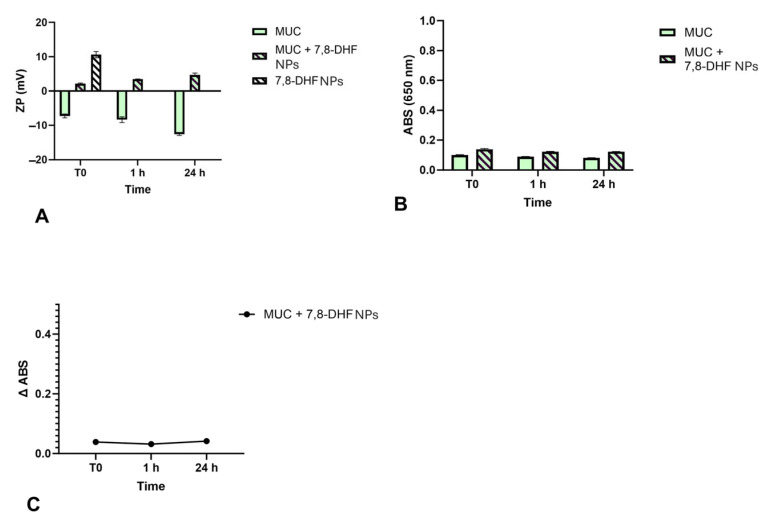
(**A**) Zeta potential (ZP) of MUC; MUC + 7,8-DHF NPs; and 7,8-DHF NPs alone after 0, 1, and 24 h of incubation, showing the electrostatic interactions between mucin and nanoparticles. (**B**) Absorbance at 650 nm of MUC and MUC + 7,8-DHF NPs over time, reflecting the extent of particle aggregation due to complex formation. (**C**) Variation in absorbance (ΔABS) for MUC + 7,8-DHF NPs.

Mucoadhesive properties of 7,8-DHF NPs were assessed by measuring the ZP of MUC suspensions over time, in both the absence and presence of 7,8-DHF NPs (Figure 14A). In the control MUC sample, ZP remained consistently negative over 24 h, ranging from approximately −7 mV at T0 to −13 mV at 24 h, reflecting the native anionic nature of mucin glycoproteins. Upon addition of NPs, a significant and progressive shift toward positive ZP values was observed, reaching +4.5 mV after 24 h. This marked inversion in surface charge is in line with previous studies, where mucin interaction similarly induced changes in particle size and ZP due to electrostatic complex formation and physical entanglement with the polymeric surface. The increase in ZP over time is indicative of a time-dependent association process, supporting the hypothesis of effective mucoadhesion. Such interactions are crucial for enhancing residence time at mucosal sites and improving drug bioavailability [88].

To further investigate the mucoadhesive potential of the nanocarrier system, turbidity measurements were performed by monitoring absorbance at 650 nm over time (Figure 14B). In the control MUC dispersion, the absorbance remained relatively low and decreased slightly over 24 h, consistent with sedimentation or structural rearrangement of mucin chains. In contrast, the MUC + NPs sample exhibited a higher absorbance at T0, which was maintained over time. This increase in turbidity suggests the formation of mucin–NP complexes, likely due to electrostatic interactions and physical entanglement between the mucin glycoproteins and the nanoparticle surface. The relatively stable absorbance values at 1 h and 24 h further indicate that the interaction leads to a persistent structural association rather than a transient effect (Figure 14C).

**Figure A3:**
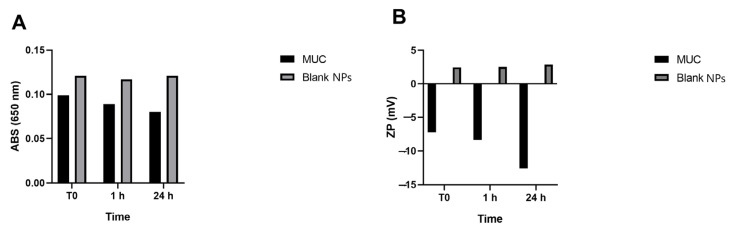
(**A**) Absorbance at 650 nm of MUC and MUC + blank NPs over time, reflecting the extent of particle aggregation due to complex formation; (**B**) zeta potential (ZP) of MUC and MUC + blank NPs after 0, 1, and 24 h of incubation, showing the electrostatic interactions between mucin and NPs.

A blank NP condition was included as a control. One-way ANOVA with Tukey’s post hoc test revealed no significant differences between blank and drug-loaded NPs (*p* > 0.05). Data are reported in Appendix A (Figure A3).

#### 3.4.7. Cell Studies

Concentration-dependent cytotoxicity of 7,8-DHF has been previously reported in neuronal and non-neuronal cell models, particularly at micromolar ranges, where prooxidant effects and mitochondrial perturbations may emerge despite its antioxidant properties [83,84,89,90]. Such dual-redox behavior is a recognized feature of catechol-containing flavonoids, which can undergo autooxidation and generate reactive intermediates under certain conditions [90]. In the present study, we compared the cytocompatibility profile of free 7,8-DHF and its nanoparticle formulation in OECs [55].

**Figure 15:**
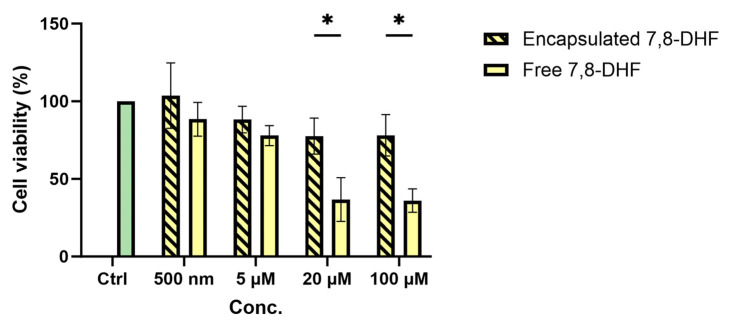
Effect of free and encapsulated 7,8-DHF on cell viability. Cells were treated with increasing concentrations of free or encapsulated 7,8-DHF (500 nM, 5 µM, 20 µM, and 100 µM), and cell viability was evaluated by MTT assay. Data are expressed as percentage of control and reported as mean ± SEM. Statistical analysis was performed using an ordinary two-way ANOVA with formulation and concentration as independent factors, including their interaction, followed by Holm–Šídák multiple-comparison tests to compare free versus encapsulated 7,8-DHF at the same concentration. * *p* < 0.05 versus free 7,8-DHF at the corresponding concentration.

**Figure A4:**
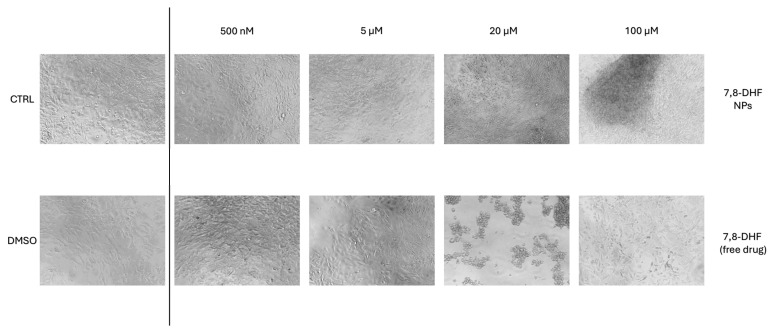
Morphological analysis of OECs following 24 h of exposure to free 7,8-DHF or 7,8-DHF NPs at increasing concentrations (500 nM, 5 µM, 20 µM, and 100 µM). Representative inverted microscopy images are shown for each condition.

Cell viability was assessed 24 h after exposure to encapsulated (7,8-DHF NPs) or free 7,8-DHF at increasing concentrations (500 nM, 5 µM, 20 µM, and 100 µM). Two-way ANOVA revealed significant effects of concentration (*p* < 0.0001) and formulation (*p* < 0.0001), as well as a significant interaction between the two factors (*p* = 0.0015). Post hoc Holm–Šídák multiple comparisons showed that encapsulated 7,8-DHF significantly preserved cell viability compared to the free drug at 20 µM and 100 µM, whereas no significant differences were observed at lower concentrations. Whereas free 7,8-DHF elicited a clear dose-dependent decline in cell viability, the NP formulation maintained substantially higher viability across the tested concentration range (Figure 15). Morphological analysis performed after 24 h of treatment showed that exposure to 7,8-DHF NPs preserved the cell monolayer integrity and typical elongated morphology across all tested concentrations up to 100 µM, indicating a favorable in vitro biocompatibility profile. At 100 µM, NPs may undergo physical agglomeration and sedimentation. The localized deposition of these aggregates onto the cell monolayer likely explains the partial reduction in cell viability. This effect can be attributed to physical shielding, where the precipitate layer restricts nutrient and oxygen diffusion led to an intensely high localized concentration of released drug directly at the cell–substrate interface. The free drug induced notable dose-dependent changes in the cellular layout. While cells treated with 500 nM of free 7,8-DHF remained comparable to the controls, treatments at 20 µM resulted in progressive monolayer disruption, characterized by cell rounding and the formation of floating cellular aggregates. At the highest free drug concentration (100 µM), a pronounced reduction in visible cell density was observed, with a loss of the continuous confluent monolayer characteristic of the control groups (Appendix A, Figure A4). The improved cytocompatibility observed with the NP formulation may be attributed to the different mode of cellular interaction compared to the free drug. While free 7,8-DHF, administered as a solution, is immediately available at the extracellular level at its full nominal concentration, potentially generating rapid concentration peaks that trigger prooxidant effects at micromolar ranges, the NP formulation presents the drug in a nanostructured form whose release follows a diffusion-based mechanism through the lipid–polymer matrix (Higuchi model). Although the NPs showed a faster initial release compared to the free drug in the dialysis-based in vitro release study, this behavior reflects drug availability in a bulk aqueous medium and may not fully replicate the in vivo scenario of nanoparticle–cell interactions. Similar findings have been reported for other bioactive compounds, where encapsulation within biodegradable polymeric carriers attenuated cytotoxic effects on normal cells compared to the free drug [91,92]. We acknowledge, however, that these interpretations remain speculative in the absence of dedicated cellular uptake studies, and future work will include fluorescence-based tracking and mechanistic investigation of the cytoprotective effect of the 7,8-DHF NP formulation.

## 4. Conclusions

In this study, 7,8-DHF was successfully encapsulated in NPs comprising natural raw materials. A DoE approach was employed to systematically optimize formulation parameters, resulting in a nanosystem with acceptable encapsulation efficiency and physicochemical properties suitable for potential nose-to-brain delivery, with dimensions tuned to favor olfactory uptake.

The optimized formulation exhibited excellent stability (Z-Ave, PDI) for up to 90 days and remained almost unchanged in simulated physiological fluids.

Compared with the free drug, 7,8-DHF NPs showed enhanced ROS scavenging activity (DPPH assay) due to the synergistic effect of the excipient matrix and drug, as well as the lower cytotoxicity in OECs, suggesting improved functional performance and cytocompatibility.

By encapsulating this lipophilic compound in a synergistic-effect matrix with good cytocompatibility, this work paves the way for future investigations on the therapeutic potential of 7,8-DHF-loaded NPs in neurodegenerative and metabolic disorders.

## Figures and Tables

**Table 1:** Values of variables in 2^3^ factorial design.

Coded Values	Actual Values		
	A = 7,8-DHF (µg)	B = Surfactant	C = Lecithin (mg)
+1	500	Pluronic^®^ F127	5
−1	100	Tween^®^ 80	10

**Table 2:** Experimental design layout of 2^3^ full factorial design.

Trials	Variable Level in Coded Form		
	A	B	C
1	−1	{−1}	−1
2	+1	{−1}	−1
3	−1	{+1}	−1
4	+1	{+1}	−1
5	−1	{−1}	+1
6	+1	{−1}	+1
7	−1	{+1}	+1
8	+1	{+1}	+1

**Table 3:** Optimization of formulation criteria by 2^3^ full factorial design.

Factors	Level	
	Lower (Unit)	Upper (Unit)
X_1_: Drug	100 μg	500 μg
X_2_: Surfactant	Pluronic^®^ F127	Tween^®^ 80
X_3_: Lecithin	5 mg	10 mg
**Responses**	**Desirability constraint**	
Y_1_: Particle size	In range	
Y_2_: PDI	In range	
Y_3_: ZP	Maximize	
Y_4_: Encapsulation efficiency	Maximize	

**Table 4:** Experimental arrangement based on the 2^3^ full factorial design and measured responses for eight experiment runs.

Code	Experimental Arrangement			Y_1_	Y_2_	Y_3_	Y_4_
	Lecithin (mg)	Surfactant Type	Drug Amount (µg)	Z-Ave (nm) ± SD	PDI ± SD	ZP ± SD	EE%
STD1	5	Tween^®^ 80	100	207.4 ± 5.9	0.158 ± 0.022	10.20 ± 1.86	7.19
STD2	5	Tween^®^ 80	500	218.8 ± 6.6	0.146 ± 0.016	8.12 ± 0.99	0
STD3	5	Pluronic^®^ F127	100	224.0 ± 3.5	0.198 ± 0.022	5.78 ± 0.31	42.89
STD4	5	Pluronic^®^ F127	500	236.5 ± 1.6	0.197 ± 0.023	11.90 ± 0.72	61.24
STD5	10	Tween^®^ 80	100	209.8 ± 4.1	0.177 ± 0.014	8.20 ± 1.32	41.40
STD6	10	Tween^®^ 80	500	222.5 ± 5.8	0.170 ± 0.011	9.85 ± 2.14	4.04
STD7	10	Pluronic^®^ F127	100	216.4 ± 3.1	0.179 ± 0.003	5.40 ± 0.17	18.60
STD8	10	Pluronic^®^ F127	500	205.7 ± 3.5	0.187 ± 0.040	5.67 ± 0.71	10.94

**Table 5:** Point prediction.

Response	Predicted Mean	Std Dev	n	95% PI Low	Data Mean	95% PI High	Error
Size	232.34	8.11	4	209	220.63	255.68	5.31
PDI	0.20	0	4	0.18	0.188	0.21	5.56
ZP	10.23	2.45	4	−0.01	7.20	20.48	42.30
EE%	58.61	1.47	4	39.57	65.23	77.66	10.10

**Table 6:** Correlation coefficient values (R^2^) of the release kinetic models.

Sample	Zero-Order	First-Order	Higuchi	Korsmeyer–Peppas
	R^2^			
7,8-DHF free drug	0.655	0.8099	0.6768	0.6145
7,8-DHF NPs	0.2778	0.6625	0.9937	0.3463

**Figure A5:**
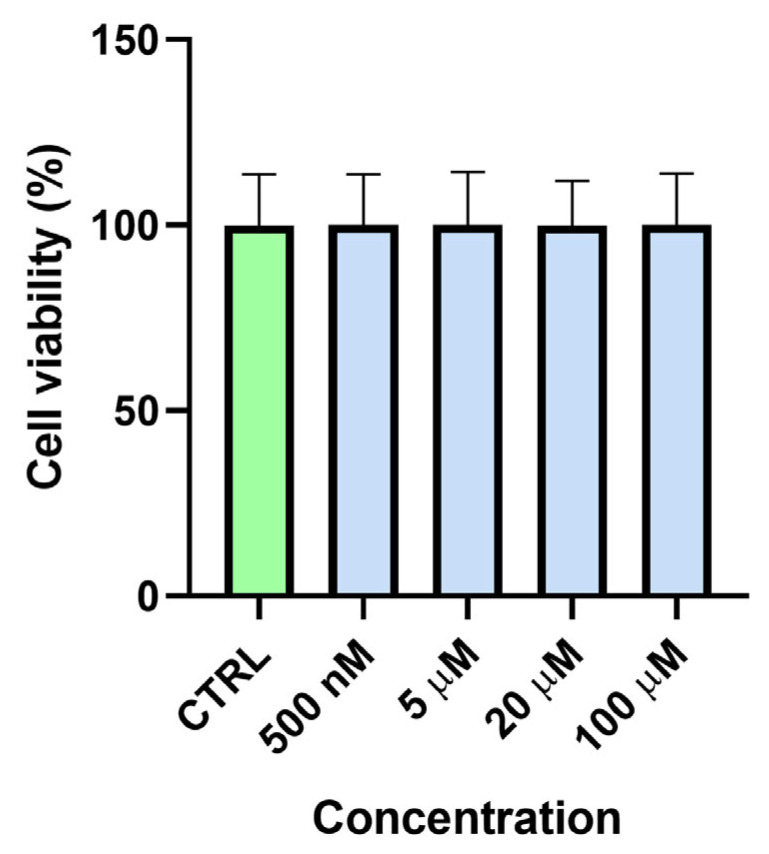
Effect of blank NPs on cell viability. Cells were treated with increasing concentrations of blank NPs corresponding to the concentration used with 7,8-DHF NPs (500 nM, 5 µM, 20 µM, and 100 µM), and cell viability was evaluated by MTT assay. Data are expressed as percentage of control and reported as mean ± SEM.

## Data Availability

The authors confirm that the data supporting the findings of this study are available on request.

## Abbreviations

The following abbreviations are used in this manuscript:

7,8-DHF	7,8-dihydroxyflavone (7,8-DHF)
aCSF	Artificial cerebrospinal fluid
ANOVA	Analysis of variance
BDNF	Brain-derived neurotrophic factor
DoE	Design of experiments
DPPH	Diphenyl-1-picrylhydrazyl
EE	Encapsulation efficiency
MUC	Mucin
NP	Nanoparticle
OEC	Olfactory ensheathing cell
PDI	Polydispersity index
SNF	Simulated nasal fluid
TEM	Transmission electron microscopy
Z-Ave	Mean particle size
ZP	Zeta potential
